# The Sur1-Trpm4 channel regulates NOS2 transcription in TLR4-activated microglia

**DOI:** 10.1186/s12974-016-0599-2

**Published:** 2016-06-01

**Authors:** David B. Kurland, Volodymyr Gerzanich, Jason K. Karimy, Seung Kyoon Woo, Rudi Vennekens, Marc Freichel, Bernd Nilius, Joseph Bryan, J. Marc Simard

**Affiliations:** Department of Neurosurgery, University of Maryland School of Medicine, 22 S. Greene St., Suite S12D, Baltimore, MD 21201-1595 USA; Department of Pathology, University of Maryland School of Medicine, Baltimore, MD USA; Department of Physiology, University of Maryland School of Medicine, Baltimore, MD USA; Department Cell Molecular Medicine, Laboratory Ion Channel Research, Campus Gasthuisberg, Herestraat 49-Bus 802, Leuven, 3000 Belgium; Pharmakologisches Institut, Universität Heidelberg, Im Neuenheimer Feld 366, Heidelberg, 69120 Germany; Pacific Northwest Diabetes Research Institute, 720 Broadway, Seattle, WA 98122 USA; Neurosurgery Research Laboratories, 10 S. Pine St, Baltimore, MD 21201-1595 USA

**Keywords:** Sur1-Trpm4, K_ATP_, NO, NOS2, TLR4, Microglia

## Abstract

**Background:**

Harmful effects of activated microglia are due, in part, to the formation of peroxynitrite radicals, which is attributable to the upregulation of inducible nitric oxide (NO) synthase (NOS2). Because NOS2 expression is determined by Ca^2+^-sensitive calcineurin (CN) dephosphorylating nuclear factor of activated T cells (NFAT), and because Sur1-Trpm4 channels are crucial for regulating Ca^2+^ influx, we hypothesized that, in activated microglia, Sur1-Trpm4 channels play a central role in regulating CN/NFAT and downstream target genes such as *Nos2*.

**Methods:**

We studied microglia in vivo and in primary culture from adult rats, and from wild type, *Abcc8*−/− and *Trpm4*−/− mice, and immortalized N9 microglia, following activation of Toll-like receptor 4 (TLR4) by lipopolysaccharide (LPS), using in situ hybridization, immunohistochemistry, co-immunoprecipitation, immunoblot, qPCR, patch clamp electrophysiology, calcium imaging, the Griess assay, and chromatin immunoprecipitation.

**Results:**

In microglia in vivo and in vitro, LPS activation of TLR4 led to de novo upregulation of Sur1-Trpm4 channels and CN/NFAT-dependent upregulation of *Nos2* mRNA, NOS2 protein, and NO. Pharmacological inhibition of Sur1 (glibenclamide), Trpm4 (9-phenanthrol), or gene silencing of *Abcc8* or *Trpm4* reduced *Nos2* upregulation. Inhibiting Sur1-Trpm4 increased the intracellular calcium concentration ([Ca^2+^]_i_), as expected, but also decreased NFAT nuclear translocation. The increase in [Ca^2+^]_i_ induced by inhibiting or silencing Sur1-Trpm4 resulted in phosphorylation of Ca^2+^/calmodulin protein kinase II and of CN, consistent with reduced nuclear translocation of NFAT. The regulation of NFAT by Sur1-Trpm4 was confirmed using chromatin immunoprecipitation.

**Conclusions:**

Sur1-Trpm4 constitutes a novel mechanism by which TLR4-activated microglia regulate pro-inflammatory, Ca^2+^-sensitive gene expression, including *Nos2*.

## Background

Toll-like receptor 4 (TLR4)-mediated neuroinflammation figures centrally in a growing list of inflammatory and degenerative conditions of the central nervous system (CNS), including traumatic brain injury, ischemic stroke, hemorrhagic stroke, Alzheimer’s disease, multiple sclerosis, Parkinson’s disease, and amyotrophic lateral sclerosis [1–4]. These conditions share the common feature that endogenous molecules associated with injury, known as alarmins or danger-associated molecular patterns (DAMPs), converge upon TLR4 and initiate potentially deleterious inflammatory cascades [2, 5, 6]. Detrimental effects of chronically activated microglia, which constitutively express TLR4 [7], have been identified in these conditions [8–11]. The ability to inhibit pro-inflammatory actions of microglia following chronic TLR4 activation has the potential to transform the treatment of a variety of degenerative CNS diseases.

A key element in the harmful effects of chronic microglial activation is peroxynitrite-mediated protein radical formation, which is attributable, in part, to de novo upregulation of microglial inducible nitric oxide (NO) synthase (NOS2) [12, 13]. Notably, NOS2 expression in various cell types is dichotomously determined by two factors whose activities are regulated by the concentration of intracellular calcium ([Ca^2+^]_i_): (i) calcineurin, the Ca^2+^-sensitive phosphatase that, by dephosphorylating nuclear factor of activated T-cells (NFAT), promotes its nuclear translocation to induce *Nos2* gene expression [14, 15] and (ii) Ca^2+^/calmodulin protein kinase II (CaMKII), the Ca^2+^-sensitive kinase that, by phosphorylating calcineurin, inhibits its phosphatase activity, thereby preventing NFAT nuclear translocation and *Nos2* gene expression [16, 17]. Thus, mechanisms regulating [Ca^2+^]_i_ may be crucial for nitrosative injury induced by activated microglia [18–20].

Ca^2+^ influx via the microglial plasma membrane can occur by multiple mechanisms, including [21] (i) voltage-operated Ca^2+^ entry (VOCE) channels, which are activated by depolarization of the plasma membrane; (ii) store-operated Ca^2+^ entry (SOCE) channels, which are opened upon depletion of intracellular Ca^2+^ stores; and (iii) receptor-operated Ca^2+^ entry (ROCE) channels, which are triggered by extracellular ligand binding events. Available data on VOCE channels in microglia are limited [20, 22]. In “electrically non-excitable” cells that do not generate all-or-none action potentials, such as microglia [19, 23], SOCE and ROCE channels serve as the major routes of Ca^2+^ entry [18–21, 24, 25].

The entry of Ca^2+^ into a cell is governed by the electrochemical gradient for Ca^2+^, with the electrical gradient being determined by the cell membrane potential [26]. Sulfonylurea receptor 1 (Sur1)-regulated ion channels have been shown to play critical roles as negative regulators of Ca^2+^ influx. In cells that utilize VOCE channels, the opening of Sur1-Kir6.2 (ATP-sensitive potassium channel (K_ATP_)) channels hyperpolarizes the cell, thereby inactivating VOCE channels and reducing Ca^2+^ influx [27]. Conversely, in cells that utilize predominantly non-voltage-operated SOCE and ROCE channels, such as microglia [20], the opening of transient receptor potential melastatin 4 (Trpm4) or Sur1-Trpm4 channels depolarizes the cell, thereby reducing the inward driving force for Ca^2+^ [26, 28–31]. Notably, Trpm4 and Sur1-Trpm4 channels are activated by intracellular Ca^2+^, with a rise in [Ca^2+^]_i_ linked directly to membrane depolarization, thereby providing negative feedback to Ca^2+^ entry through SOCE or ROCE channels [26].

Recent evidence indicates that Sur1 inhibition results in robust anti-inflammatory effects in CNS injury. In models of cerebral ischemia and spinal cord injury, glibenclamide inhibition of Sur1 is associated with enhanced microglial phagocytosis and improved neurological function, with these effects attributed to inhibition of microglial Sur1-Kir6.2 (K_ATP_) channels [32–35]. In models of subarachnoid hemorrhage and multiple sclerosis, gene suppression or pharmacological inhibition (glibenclamide) of *Abcc8*/Sur1 significantly ameliorates neuroinflammation and improves neurological function, with these effects attributed to inhibition of Sur1-Trpm4 channels [36–38]. Importantly, inhibition of *Abcc8*/Sur1 does not distinguish between Sur1-Kir6.2 (K_ATP_) and Sur1-Trpm4 channels.

Here, we hypothesized that microglia activated by TLR4 ligation upregulate Sur1-Trpm4 channels and that, in TLR4-activated microglia, Sur1-Trpm4 channels play a central role in regulating [Ca^2+^]_i_ and thus the expression of Ca^2+^-sensitive genes such as *Nos2*.

## Methods

### Reagents

Lipopolysaccharide (LPS) [from *E. coli* R515 (Re), TLR*grade*™] and FK506 were purchased from Enzo Life Sciences (Farmingdale, NY, USA). Papain, dispase II, glibenclamide, 9-phenanthrol, diazoxide, SKF-96365, A23187, 1,2-bis(2-Aminophenoxy)ethane-N,N,N′,N′-tetraacetic acid acetoxymethyl ester (BAPTA-AM), KN-93, and Percoll were purchased from Sigma-Aldrich (St. Louis, MO, USA). 11R-VIVIT was purchased from EMD Millipore (Billerica, MA, USA). The TLR4 signaling inhibitor, TAK-242, was purchased from Invivogen (San Diego, CA, USA). Artificial cerebrospinal fluid (aCSF) was purchased from Tocris Bioscience (Avonmouth, Bristol, UK). All culture media, sera, antibiotics, DNase I, Fluo-4-AM, and pluronic were obtained from Thermo Fisher Scientific (Waltham, MA, USA). All drugs and Fluo-4-AM were solubilized in dimethylsulfoxide (DMSO) vehicle. Papain, dispase II, and DNase I were solubilized in culture media. Sera and antibiotics were added directly to culture media.

### Animals and surgical procedure

We certify that all applicable institutional and governmental regulations concerning the ethical use of animals were followed during the course of this research. Animal experiments were performed under a protocol approved by the Institutional Animal Care and Use Committee (IACUC) of the University of Maryland, Baltimore and in accordance with the relevant guidelines and regulations as stipulated in the United States National Institutes of Health Guide for the Care and Use of Laboratory Animals. All efforts were made to minimize the number of animals used and their suffering.

Prior to surgery, sterile mini-osmotic pumps (1007D, 0.5 μL/h; Alzet, DURECT Corporation, Cupertino, CA, USA) were loaded per the manufacturer’s instructions with sterile normal saline (NS; Quality Biological Inc., Gaithersburg, MD, USA) for sham controls, or 0.416 mg/mL LPS diluted in NS, to deliver 5 μg/day LPS. The pumps were attached to sterile brain infusion kits (Alzet; Brain Infusion Kit 2). Male Wistar rats aged 8–12 weeks (Harlan, Indianapolis, IN, USA) were anesthetized (60 mg/kg ketamine plus 7.5 mg/kg xylazine, immunoprecipitation (IP)) and allowed to breathe air spontaneously. Body temperature was measured rectally and maintained throughout surgery at 37 ± 1 °C using a heating pad (Harvard Apparatus, Holliston, MA, USA). Surgical incision sites were prepared using iodine and alcohol, and a sterile environment was maintained throughout the procedure. Rats were mounted in a stereotactic apparatus (Stoelting Co., Wood Dale, IL, USA). A midline scalp incision was made to expose the skull. A 1-mm burr hole was made over the right striatum [AP, +0.75 mm; ML, +1.7 mm relative to bregma], and the dura was opened sharply. A pre-loaded mini-osmotic pump was attached to a brain infusion kit, and the needle was advanced through the burr hole to a final depth of 5 mm under stereotaxic guidance. Cyanoacrylate glue was used to secure the applicator to the dorsal surface of the skull.

Wild-type (WT) male C57BL/6J mice were obtained from The Jackson Laboratory (Bar Harbor, ME, USA). Male *Abcc8−/−* and *Trpm4*−/− mice were obtained as described previously [39, 40]. Mice were housed under pathogen-free conditions in the animal facility of the University of Maryland School of Medicine. Mice were anesthetized (60 mg/kg ketamine plus 7.5 mg/kg xylazine, IP) and allowed to breathe room air spontaneously. Body temperature was measured rectally and maintained throughout surgery at 37 ± 1 °C using a heating pad (Harvard Apparatus, Holliston, MA, USA). All surgical incision sites were prepared with iodine and alcohol, and a sterile environment was maintained during surgical procedures. Mice were mounted in a stereotactic apparatus (Stoelting Co.). A midline scalp incision was made to expose the skull. A 1-mm burr hole was made over the right striatum (AP, +1 mm; ML, +1.5 mm; DV, −2 mm relative to the bregma), and the dura was opened sharply. A pre-loaded neurosyringe (Stoelting Co.), mounted on the stereotactic frame and containing either sterile aCSF or LPS (0.1 μg/μL) in aCSF, was advanced to the final coordinates. Solution (5 μL) was infused slowly over 5 min. The syringe was left in place for an additional 5 min to minimize backflow and then was removed prior to sterile wound closure.

Animals were euthanized by IP injection of pentobarbital (>100 mg/kg), followed by perfusion of NS intracardially. For microglia, RNA, and protein isolation, brains were rapidly harvested and processed using standard techniques, described below. For mice whose brains were to be used for histology, NS perfusion was followed by perfusion with 10 % neutral buffered formalin. The brain was removed, immersion fixed 24 h in formalin, and cryoprotected 48 h in 30 % sucrose prior to cryosectioning.

### In situ hybridization

Digoxigenin (DIG)-labeled probes (Integrated DNA Technologies, Coralville, IA, USA) were designed to hybridize to nucleotides located within coding sequences of rat *Abcc8, Trpm4*, and *Kcnj11* genes. The following antisense sequences were used as probes: *Abcc8:* 5′-GCCCGGGCACCCTGCTGGCTCTGTGTGTCCTTCCGCGCCTGGGCATCG-3′; *Trpm4:* 5′-CCAGGGCAGGCCGCGAATGGAATTCCCGGATGAGGCTGTAGCGCTGCG-3′; and *Kcnj11:* 5′-GCCACTTGAGGTCCACCAGCGTGGTGAACA-3’. Corresponding sense sequences were used as negative controls. In situ hybridization (ISH) was performed on 10-μm-thick sections on glass slides using an ISH Kit (Biochain Institute, Inc., Newark, CA, USA) according to the manufacturer’s protocol. Sections were washed twice with DEPC-PBS and then were treated with 10 μg/mL proteinase K at 37 °C for 10 min. Slides were washed in DEPC-PBS, rinsed with DEPC-H_2_O, and pre-hybridized with ready-to-use pre-hybridization solution (BioChain Institute) for 3 h at 50 °C. The DIG-labeled probes were diluted in hybridization buffer (BioChain Institute) and applied at 4 ng/μL. Sections were incubated at 45 °C for 16 h. Post-hybridization washing and immunological detection, using anti-DIG-HRP and Tyramide Signal Amplification with cyanine 3 (TSA™-Cy3; Perkin Elmer, Waltham, MA, USA), were performed as recommended by the manufacturer. Finally, slides were rinsed in distilled H_2_O and then immunolabeled for P2Y12 using a fluorescent secondary antibody (Alexa Fluor 488), as described below. The red fluorescence indicates *Abcc8*, *Trpm4*, or *Kcnj11* mRNA; green fluorescence indicates immunohistochemical staining for microglia.

Unbiased measurements of signal intensity within regions of interest (ROIs) were obtained using NIS-Elements AR software (Nikon Instruments, Melville, NY, USA). The area that was evaluated was a square, 1000 × 1000 μm, centered on the tip of the needle track in the striatum, in the coronal section 200 μm rostral to the site of injection. The pixels occupied by specific P2Y12 labeling (>2× background) within this square were defined as the ROI. Specific labeling for *Abcc8*, *Trpm4*, or *Kcnj11* within the ROI was defined as pixels with signal intensity greater than twice that of the background. Specific ISH labeling within the ROI was normalized to saline-injected control. Results, expressed as fold change in the microglial expression of mRNA, were obtained from five independent experiments.

### Immunofluorescence labeling

Coronal cryosections (10 μm) on glass slides were blocked (5 % goat or 2 % donkey serum, + 0.2 % Triton X-100 for 1 h at room temperature) and then incubated overnight at 4 °C with primary antibodies. After several rinses in phosphate-buffered saline, the slides were incubated for 1 h with fluorescent-labeled species-appropriate secondary antibodies (1:500; Alexa Fluor 488 and Alexa Fluor 555; Invitrogen, Molecular Probes, Eugene, OR, USA) at room temperature. Omission of primary antibody was used as a negative control. The sections were coverslipped with polar mounting medium containing antifade reagent and 4′,6-diamidino-2-phenylindole (DAPI; Invitrogen, Eugene, OR, USA) and were examined using epifluorescence microscopy (Nikon Eclipse 90i; Nikon Instruments Inc., Melville, NY, USA). Immunofluorescent labeling of microglial cells cultured on glass chamber slides was carried out similarly, following a 15-min fixation in 4 % paraformaldehyde.

The following primary antibodies were used: goat anti-ionized Ca^2+^-binding adapter molecule 1 (Iba1) (1:1,000; Wako Chemicals, Richmond, VA, USA); rabbit anti-P2Y12 (1:200; Anaspec, Fremont, CA, USA); mouse anti-ED1 (1:500; EMD Millipore); rabbit anti-Sur1 (1:200, custom [41]); rabbit anti-Trpm4 (1:200, custom [41]); goat anti-Kir6.2 (1:200, G-16, Santa Cruz); and mouse anti-NFATc1 (1:200, Santa Cruz).

For quantitative immunohistochemistry, all tissue and cells were immunolabeled as a single batch, and all images were collected using uniform parameters of magnification and exposure, as previously described [37]. Unbiased measurements of signal intensity within ROIs were obtained using NIS-Elements AR software (Nikon Instruments). Segmentation analysis was performed by computing a histogram of pixel intensity for a particular ROI. Quantification of microglial expression of Sur1, Trpm4, and Kir6.2 in vivo was performed as described above for ISH, using P2Y12 immunolabeling as the ROI.

For quantification of nuclear translocation of NFATc1 in vitro, the nuclei of 50 cells or more were analyzed, with the ROI defined by DAPI labeling. Specific labeling for NFATc1 within the ROI was defined as pixels with signal intensity greater than twice that of the background. Specific labeling within the ROI was normalized to DAPI. Results were obtained from five independent experiments.

### Isolation and culture of primary adult microglia

A highly enriched population of microglia was isolated by Percoll density centrifugation from adult rat brains and adult WT, *Abcc8−/−*, and *Trpm4−/−* mouse brains using a protocol described previously [42]. Briefly, an adult rat or mouse was perfused with ice-cold saline and the intact brain was collected and placed onto a 35-mm dish in 2-mL ice-cold serum free cell culture medium. The brain was finely minced with a razor blade, transferred to a 15-mL tube containing 3 mL of dissociation medium [papain (1 mg/mL), dispase II (1.2 U/mL), and DNase I (20 U/mL) in serum free medium] and incubated at 37 °C with constant agitation for 30 min. The enzymes were neutralized by adding 5 mL of culture medium containing serum, and debris was removed by 5-min centrifugation at 250×*g*, followed by a resuspension of the pellet in serum-free medium. Following gentle trituration, the cell suspension was filtered sequentially through 100-, 70-, and 40-μm mesh cell strainers (Thermo Fisher Scientific). Debris was removed by 5-min centrifugation at 250×*g*, and the pellet was resuspended in 4 mL 37 % standard isotonic Percoll (SIP). Homogenized brain tissue suspended in 37 % SIP was transferred to a new 15-mL tube, underlaid with 4 mL 70 % SIP and overlaid with 4 mL 30 % SIP following by 2 mL of Hank’s balanced salt solution (HBSS). Following centrifugation at 300×*g* for 40 min at 18 °C, a distinct interphase layer containing microglia could be observed. This layer was carefully removed and washed by centrifugation twice, as described above.

The purity of isolated cells was determined by quantitative real-time polymerase chain reaction (qPCR). Microglia from one rat were suspended in Dulbecco’s modified Eagle’s medium (DMEM)/F12 plus 10 % fetal bovine serum (FBS) to a concentration of 5 × 10^5^ cells/mL and plated onto 6-well culture dishes (Corning). Microglia from one mouse were suspended in DMEM/F12 plus 10 % FBS to a concentration of 5 × 10^5^ cells/mL and plated onto two wells of a 96-well plate (Corning) to allow for paired analysis of control versus LPS treatment conditions from one animal. All experiments with primary microglia were begun following an overnight incubation at 37 °C with 5 % CO_2_.

### RNA isolation and quantitative real-time polymerase chain reaction

The MIQE guidelines [43] were consulted for the preparation, handling, and analysis of qPCR samples. Microglial cells were homogenized in Trizol Reagent (Thermo Fisher Scientific), and total RNA was isolated with Direct-zol™ RNA MiniPrep Kit (Zymo Research; Irvine, CA, USA). To avoid contamination by genomic DNA, RNA was further purified with Amplification Grade DNase I (Invitrogen). The concentration of total RNA was determined by measuring the optical density at 260 and 280 nm. The quality of RNA was evaluated using an Agilent Bioanalyzer (Agilent Technologies; Santa Clara, CA, USA); samples with an RNA integrity number (RIN) <7 were excluded from analysis.

cDNA was synthesized from 1 μg of total RNA of each sample using SuperScript III Reverse Transcriptase (RT) Supermix (Thermo Fisher Scientific). Generated cDNAs were stored at −20 °C. qPCR reactions (25 μL), consisted of 1 μL cDNA template, Platinum SYBR Green SuperMix-UDG with ROX (2× concentrated, Thermo Fisher Scientific), specific primers, and ultra-pure H_2_O. The abundance of various mRNA in the samples was determined by qPCR (ABI PRISM 7300; Applied Biosystems, Carlsbad, CA, USA). Reactions were incubated at 50 °C for 2 min and 95 °C for 2 min, followed by 40 cycles of 95 °C for 15 s and 60 °C for 30 s, followed by melting curve analysis. No-template and no-RT reactions were used as negative controls in every experiment. The absence of PCR inhibitors in the reactions was determined using the Alien Reference RNA qPCR Detection Kit (Agilent Technologies). *Rps18* and glyceraldehyde 3-phosphate dehydrogenase (*Gapdh*) mRNA were measured as reference genes to normalize the samples. The primers used in this study are listed in Table 1. Melting curve analysis was used to confirm the validity of experimental results.

**Table 1:** Primers used for qPCR in this study

Gene name	Species	Sequence accession number	Primer sequence	Amplicon length
*Abcc8*	Rat	NM_013039.2	5′-TCATCCGGGTGAGGAGATAC-3′ (+)	130
			5′-CACCAGTAGGTCCCCTTTGA-3′ (−)	
*Trpm4*	Rat	NM_001136229.1	5′-GCAAGTTCTGAGGACTCTGTTG-3′ (+)	140
			5′-TTGCATCCTGTTGCATGTTGGC-3′ (−)	
*Kcnj11*	Rat	NM_031358.3	5′-TGCGTCACAAGCATCCACTCCT-3′ (+)	100^a^
			5′-GGACATTCCTCTGTCACCATGC-3′ (−)	
*Kcnj8*	Rat	NM_017099.4	5′-CACTTCGGGAGGTCTCTGC-3′ (+)	69
			5′-GCGTCCTCCTAGAAGACTCGG-3′ (−)	
*Il-1β*	Rat	NM_031512.2	5′-AAATGCCTCGTGCTGTCTGA-3′ (+)	85
			5′-TGGAGAATACCACTTGTTGGC-3′ (−)	
*P2y12*	Rat	NM_022800.1	5′-CTTTGGCAACGAAACCAAGT-3′ (+)	127
			5′-CACCTCCATGGTCCTGGTTA-3′ (−)	
*Tlr4*	Rat	NM_019178.1	5′-TCATGCTTTCTCACGGCCTC-3′ (+)	142
			5′-AGGAAGTACCTCTATGCAGGGAT-3′ (−)	
*Gfap*	Rat	NM_017009.2	5′-CCAGATCCGAGAAACCAGCC-3′ (+)	88
			5′-CCGCATCTCCACCGTCTTTA-3′ (−)	
*Neun*	Rat	NM_001134498.2	5′-CGCAGCCTACAGTGACAGTTAT-3′ (+)	132
			5′-GTGAAGCGGCTGTACCCTC-3′ (−)	
*Gapdh*	Rat	NM_017008.4	5′-CATCACTGCCACTCAGAAGACTG-3′ (+)	153^b^
			5′-ATGCCAGTGAGCTTCCCGTTCAG-3′ (−)	
*Abcc8*	Mouse	NM_011510.3	5′-GCCAGCTCTTTGAGCATTGG-3′ (+)	102
			5′-AGGCCCTGAGACGGTTCTG-3′ (−)	
*Trpm4*	Mouse	NM_175130.4	5′-TGTTGCTCAACCTGCTCATC-3′ (+)	83
			5′-GCTGTGCCTTCCAGTAGAGG-3′ (−)	
*Kcnj11*	Mouse	NM_010602.3	5′-TGCGTCACAAGCATCCACTCCT-3′ (+)	100^c^
			5′-GGACATTCCTCTGTCACCATGC-3′ (−)	
*Il-6*	Mouse	NM_031168.2	5′-CCCCAATTTCCAATGCTCTCC-3′ (+)	141
			5′-CGCACTAGGTTTGCCGAGTA-3′ (−)	
*Nos2*	Mouse	NM_010927.4	5′-TGGAGCGAGTTGTGGATTGTC-3′ (+)	98
			5′-GGGCAGCCTCTTGTCTTTGA-3′ (−)	
*Fth1*	Mouse	NM_010239.2	5′-CGAGATGATGTGGCTCTGAA-3′ (+)	94
			5′-TCTGCAGCTTCATCAGTTTCTC-3′ (−)	
*Cd11b*	Mouse	NM_001082960.1	5′-AAGGATTCAGCAAGCCAGAA-3′ (+)	100
			5′-TACTCTTCAGAGCCCCATGC-3′ (−)	
*Gfap*	Mouse	NM_001131020.1	5′-TGCTGGAGGGCGAAGAAAACCG-3′ (+)	83
			5′-TTTGGTGCTTTTGCCCCCTCGG-3′ (−)	
*Neun*	Mouse	NM_001039167.1	5′-GTTGCCTACCGGGGTGCACAC-3′ (+)	110
			5′-TGCTCCAGTGCCGCTCCATAAG-3′ (−)	
*Rps18*	Mouse	NM_011296.2	5′-CGGAAAATAGCCTTCGCCATCAC-3′ (+)	134
			5′-ATCACTCGCTCCACCTCATCCT-3′ (−)	
*Gapdh*	Mouse	NM_008084.3	5′-CATCACTGCCACCCAGAAGACTG-3′ (+)	153^d^
			5′-ATGCCAGTGAGCTTCCCGTTCAG-3′ (−)	

For *Kcnj11* and *Gapdh*, same primers used for rat and mouse

^a^ Amplicon is from 564 to 664

^b^ Amplicon is from 609 to 761

^c^ Amplicon is from 668 to 768

^d^ Amplicon is from 584 to 736

### Patch clamp electrophysiology

Patch clamp electrophysiology was performed as described [41, 44, 45]. Whole cell recordings were performed using a nystatin perforated patch technique, to minimize the disturbance of the intracellular mileu that causes rapid rundown of Trpm4 currents [46, 47]. Nystatin, 50 mg, (Calbiochem, San Diego, CA, USA) was dissolved in DMSO, 1 ml. Working solutions were made before the experiment by adding 16.5 μL nystatin stock solution to 5 mL of the base pipette solution to yield a final concentration of nystatin of 165 μg/mL and DMSO 3.3 μL/ml.

To record whole cell macroscopic currents under “physiological” conditions, the extracellular solution contained (mM) NaCl 130, KCl 10, CaCl_2_ 1, MgCl_2_ 1, HEPES 32.5, glucose 12.5, and pH 7.4 and the pipette solution contained (mM) KCl 55, K_2_SO_4_ 75, MgCl_2_ 8, and HEPES 10, and nystatin, 165 μg/mL, pH 7.2.

To record whole cell macroscopic currents exclusive of K^+^ channels, the extracellular solution contained (mM) CsCl 145, CaCl_2_ 1, MgCl_2_ 1, HEPES 32.5, glucose 12.5, and pH 7.4 and the pipette solution contained (mM) CsCl 145, MgCl_2_ 8, and HEPES 10, and nystatin, 165 μg/mL, pH 7.2.

The following parameters were used: holding potential, −50 mV; ramp pulses were from −100 to +100 mV, 4 mV/msec, applied every 15 s.

Steady-state inward currents were quantified at −50 mV and are presented in bar graphs as positive values, normalized to cell capacitance.

### Cell culture

The N9 murine microglial cell line (Neuro-Zone, Milan, Italy) was cultured in Iscove’s modified Dulbecco’s medium (IMDM) with 5 % FBS. N9 cells were seeded at 1.5 × 10^5^ cells/mL and allowed to adhere overnight prior to experimental manipulation. LPS was used at a final concentration of 1 μg/mL to activate TLR4; all experiments with LPS were performed in 5 % FBS. Glibenclamide (30 μM), diazoxide (100 μM), 9-phenanthrol (5 μM), A23187 (1 μM), BAPTA-AM (10 μM), SKF-96395 (7.5 μM), TAK-242 (3 μM), FK506 (1 μM), and 11R-VIVIT (10 μM), KN-93 (3 μM), all dissolved in DMSO, were used at final concentrations indicated, and were added concurrently with LPS for immunofluorescence and immunoblot experiments. Reagents were added at the time of recording for Ca^2+^ imaging experiments. Cultured cells were maintained 37 °C and 5 % CO_2_.

All cell culture experiments were carried out in the presence of 5 % FBS. Glibenclamide is reported to be 99 % protein bound [48], indicating that the free concentration of drug would be much less than that the apparent concentration that was added. We independently verified the reported degree of protein binding using a method that we previously described for measuring free drug concentration [49]. Briefly, various amounts of a stock solution of glibenclamide (25 mg per mL of DMSO) were added to NS containing 5 % FBS, and the solution was dialyzed against NS (Mini Slide-A-Lyzer, 3.5 K MWCO; Thermo Fisher Scientific). The concentration of glibenclamide in the dialysate was measured spectrophotometrically (absorbance at 239 nm), and the final concentration was determined using a standard curve that we constructed. For each concentration, dialysis reactions with vehicle were performed to control for the background. Linear fit of data at different concentrations of glibenclamide showed that drug was 98.8 % protein bound.

### Immunoprecipitation and immunoblotting

For immunoprecipitation experiments, total lysate from N9 cells was prepared in 3-[(3-cholamidopropyl)dimethylammonio]-1-propanesulfonate (CHAPS) lysis buffer (pH 8.0; FivePhoton Biochemicals, San Diego, CA, USA) supplemented with freshly added protease and phosphatase inhibitor cocktail (PPI, Cell Signaling Technology, Danvers, MA, USA) and spermidine (100 mM, Sigma-Aldrich). Crude lysate was homogenized by centrifugation through a Qiashredder column (2 minutes; 6,000 RPM; QIAGEN, Valencia, CA, USA), and the pellet was gently resuspended to minimize loss of hydrophobic membrane proteins. Prior to lysate collection for co-immunoprecipitation experiments, protein crosslinking was performed in cell culture dishes using 1 mM DSP (dithiobis(succinimidyl propionate); Thermo Fisher Scientific) according to the manufacturer’s instructions.

We followed our previously validated approach to evaluate the expression of Sur1 and Trpm4 by immunoblot [41]. To immunoprecipitate Sur1 or crosslink-stabilized Sur1-Trpm4, a custom goat anti-Sur1 antibody [41] was incubated with Dynabeads Protein G (Thermo Fisher Scientific) according to the manufacturer’s instructions. To immunoprecipate Trpm4, a custom chicken anti-Trpm4 antibody [41] was covalently coupled to Dynabeads M-270 Epoxy according to the manufacturer’s instructions using an antibody coupling kit (Thermo Fisher Scientific). Following a wash step, crude lysate was added to the antibody-bound magnetic beads and incubated with constant rotation overnight at 4 °C. The immune complexes formed were isolated by placing the reaction tube against a magnet and washed twice with lysis buffer. To elute the proteins and fully reduce crosslinked proteins, the beads were resuspended in a 2× LDS sample buffer with 1× reducing agent (Thermo Fisher Scientific), vortexed at full speed and then kept at 37 °C for 30 min. Following application of a strong magnet to remove the beads, the resulting samples were used directly for sodium dodecyl sulfate polyacrylamide gel electrophoresis (SDS-PAGE) and were examined by immunoblot analysis. Reactions using plain beads (lysate without addition of IP antibody) and antibody only (beads and IP antibody without the addition of lysate) were used as negative controls. In order to study individual protein expression, we performed immunoprecipitation of Sur1 followed by immunoblot of Sur1, or immunoprecipitation of Trpm4 followed by immunoblot of Trpm4. In order to study the interaction between Sur1 and Trpm4, we performed immunoprecipitation of Sur1 followed by immunoblot of Trpm4. Sur1 or Trpm4 proteins were detected using custom rabbit anti-Sur1 and rabbit anti-Trpm4 antibodies [41].

For the analysis of the subcellular localization of proteins in N9 microglia, an optimized protocol for the fractionation of cytoplasmic versus nuclear protein was performed rapidly, as follows. First, adhered cells were incubated in ice-cold hypotonic buffer containing dilute detergent (10 mM Tris, 0.1 % Triton X-100, supplemented with PPI) for 3 min. With the aid of a cell scraper, cells were collected into a 1.5-mL tube and vortexed for 3 s. The contents were immediately centrifuged for 5 min, and the supernatant containing soluble cytoplasmic proteins was collected in a new tube. The remaining pellet containing intact nuclei was then resuspended in radio-immunoprecipitation assay (RIPA) lysis buffer (Thermo Fisher Scientific) supplemented with PPI and left on ice for 10 min to allow for dissolution of nuclear membranes. Following this, a reduction in the viscosity of nuclear protein samples was carried out by homogenization through a Qiashredder column, as above. Successful fractionation was confirmed via immunoblot of the cytoplasmic protein lactate dehydrogenase (LDH) and the nuclear protein histone deacetylase 1 (HDAC1). For all other applications, unless otherwise stated, protein was harvested in RIPA lysis buffer supplemented with PPI and homogenized by centrifugation through a Qiashredder column.

The following primary antibodies were used: mouse anti-NFATc1 (1:200; 7A6, Santa Cruz); rabbit anti-phosphorylated CaMKII (Thr286, pCaMKII, 1:2,000, Cell Signaling Technology); rabbit anti-CaMKII (pan, 1:2,000, Cell Signaling Technology); mouse anti-NOS2 (1:2,000, Thermo Fisher Scientific); rat anti-HSC70 (1:10,000, Abcam, Cambridge, MA, USA); rabbit anti-phosphorylated calcineurin (Ser197, pCN, 1:200; Badrilla Ltd., Leeds, UK); rabbit anti-calcineurin (pan, 1:1000; Cell Signaling Technology); rabbit anti-LDH (1:2,000, Santa Cruz); and mouse anti-HDAC1 (1:10,000, Cell Signaling Technology). Protein was detected using species-appropriate horse radish protein-tagged secondary antibodies (Cell Signaling Technology). Detection was performed using the ECL system (Amersham BioSciences Inc., Piscataway, NJ, USA) with routine imaging (Fuji LAS-3000) and quantification (ImageJ). For NFATc1, band densities of each isoform (1-3) were combined into a single value for quantification, which was done similarly for the two major isoforms of CaMKII (α/β). Acquired data were normalized to appropriate loading controls.

### Ca^2+^ imaging

Changes in intracellular Ca^2+^ were assessed in N9 microglia using a Ca^2+^-sensitive indicator, Fluo-4-AM (Invitrogen), as previously reported [50]. Cells were cultured on 35-mm fluorodishes (World Precision Instruments; Sarasota, FL, USA). Prior to Ca^2+^ imaging experiments, the cells were incubated overnight in phenol red-free and serum-free culture medium. A stock solution containing Fluo-4-AM and the non-toxic dispersing agent, Pluronic F-127, was then added directly to the cells to final concentrations of 5 μM and 0.02 %, respectively. Loading of Ca^2+^ indicator was performed for 30 min at 37 °C. After loading, cells were gently washed with and then maintained in phenol red-free and serum-free culture medium. For imaging, loaded cells were placed in a temperature controlled chamber (37 °C) on the stage of a confocal microscope (LSM Duo, Zeiss, Germany) and allowed to equilibrate for 15 min. For each experiment, complete equilibration was confirmed by imaging for a short time series (~3 min) and observing a plateau of fluorescence signal. Using a 20× objective, Fluo-4-AM-loaded cells were excited by a HeNe Laser source with a 488 ± 10-nm excitation, and the fluorescence signal was collected at 530 ± 10-nm emission. For each experiment, images were taken every 4 s and the field was recorded for 10 mins. From each field, ten cells were chosen randomly for analysis. The changes in the fluorescence intensity within the selected cells were quantitatively analyzed using Zen software (Zeiss). Results were obtained from a minimum of three independent experiments.

### Chromatin immunoprecipitation

Chromatin immunoprecipitation (ChIP) was performed to study NFATc1 binding to the *Nos2* promoter using ChIP-IT® High Sensitivity kit (Active Motif; Carlsbad, CA, USA). Chromatin was prepared as follows, from N9 microglia. Cells were fixed, lysed, and then sonicated to fragment chromatin, according to the manufacturer’s instructions. After validating chromatin shearing efficiency via gel electrophoresis, immunoprecipitation of NFATc1-DNA complexes was performed using ChIP-validated rabbit anti-NFATc1 antibody (H-110, Santa Cruz Biotechnology). For each reaction, 4 μg DNA and 0.4 μg antibody were used, and the reactions were incubated overnight at 4 °C under constant rotation. Parallel immunoprecipitation reactions using equivalent amounts of rabbit IgG were performed as background controls. Following reversal of crosslinks and purification of DNA, qPCR was performed as described above using primers aligned with an NFAT binding site (−1000 to −877) on the *Nos2* promoter [14]. We analyzed the *Nos2* promoter using MatInspector software (Genomatix Inc.; Ann Arbor, MI, USA) and independently identified this NFAT binding site.

### MTT assay

Cell viability was measured using the Vybrant® MTT Cell Proliferation Assay Kit (Thermo Fisher Scientific). Briefly, a 12-mM MTT stock solution was prepared by adding 1 mL of sterile PBS to one 5-mg vial of MTT; 10 μL of the 12-mM MTT stock solution was added to cells cultured in 96-well plates containing 100 μL media and incubated at 37 °C for 4 h. After labeling the cells with MTT, all but 25 μL of medium was removed from the wells; 50 μL of DMSO was added to each well and mixed thoroughly, followed by an incubation at 37 °C for 10 min. Absorbance was read at 540 nm; a decrease in absorbance in treatment groups versus control indicated reduced cell viability.

### Griess assay

Nitrite was quantified from N9 microglia culture media using a Griess Reagent Kit (Thermo Fisher Scientific), according to the manufacturer’s instructions. Briefly, equal volumes of *N*-(1-naphthyl)ethylenediamine (component A) and sulfanilic acid (component B) were combined to form the Griess Reagent; 20 μL of the Griess Reagent was added to 150 μL of the nitrite containing sample, followed by the addition of 130 μL deionized water. Following a 30-min incubation at room temperature, the absorbance was measured at 548 nm. Calibrations and standard curves were generated from sodium nitrite standards (1–100 μM).

### Statistical analysis

Data are presented as mean ± standard error. Statistical comparisons were made using Student’s *t* test or analysis of variance (ANOVA), as appropriate, with post hoc comparisons made using Fisher’s method. Calculations were performed with OriginPro2016 (OriginLab Corp., Northampton, MA, USA). A value *p* < 0.05 was considered to be statistically significant.

## Results

### Sur1-Trpm4 upregulation in TLR4-activated microglia

#### TLR4 activation in vivo induces Sur1-Trpm4 channel expression in microglia

**Fig. 1:**
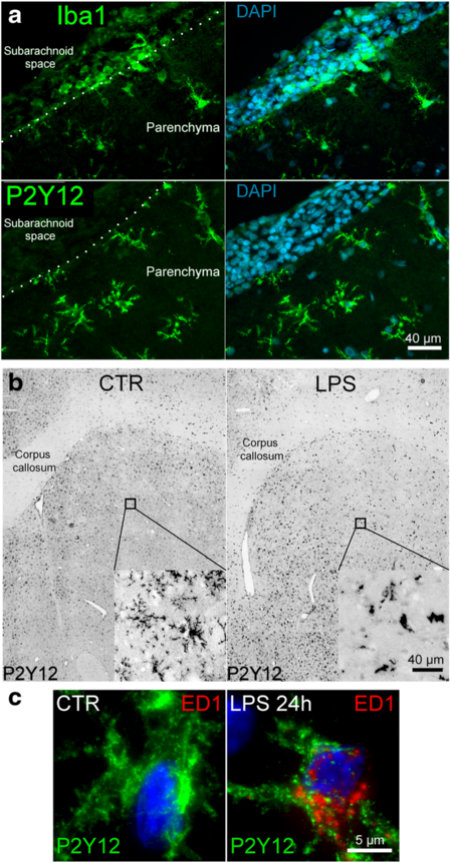
Model of TLR4 activation of rat microglia in vivo. **a** Immunofluorescence images of adjacent 10-μm coronal brain sections 6 h after intrastriatal infusion of LPS (rate, 5 μg/day); Iba1^+^ cells (*green*, *upper*) versus P2Y12^+^ cells (*green*, *lower*); *dotted lin*e demarcates the pial layer separating the subarachnoid space from the parenchyma; nuclei labeled with DAPI (*blue*); *scale bar* 40 μm. **b** *Grayscale* images of brain sections of control (CTR) versus 24 h after intrastriatal infusion of LPS (LPS 24 h); *cc* corpus callosum; P2Y12 labeling (*black*); morphological changes in activated microglia are shown in the *inset*; *scale bar* 40 μm. **c** High magnification immunofluorescence images representative of microglia under control (*CTR*) conditions versus 24 h after intrastriatal infusion of LPS (LPS 24 h); P2Y12 (*green*) and ED1 (*red*); nuclei labeled with DAPI (*blue*)

LPS infusion into the striatum of adult rats, which leads to nitrosative/oxidative stress and neuroinflammation [51], was used as a model to study TLR4 activation in vivo. Microglia were identified using various markers, including the purinergic receptor, P2Y12, which is expressed by microglia but not by infiltrating myeloid cells, and Iba1, cluster of differentiation molecule 11b (Cd11b), and cluster of differentiation molecule 68 (CD68/ED1), with the last three expressed by both microglia and myeloid cells [52]. Following TLR4 activation for 6 h in vivo, Iba1^+^ cells were localized both within the CNS parenchyma and in the subarachnoid space, consistent with both resident microglia and infiltrating monocytes (Fig. 1a, upper panels), whereas P2Y12^+^ cells were identified only in the CNS parenchyma, consistent with selective expression by microglia (Fig. 1a, lower panels). In subsequent experiments, P2Y12 immunolabeling was employed to identify microglia distinct from infiltrating myeloid cells.

TLR4 activation in vivo for 24 h led to the development of an activated microglial phenotype [53]. Activation was characterized by a morphological shift from a highly ramified appearance in quiescent, P2Y12^+^ microglia, which were ED1^−^, to an amoeboid appearance in TLR4-activated P2Y12^+^ microglia, which were ED1^+^ (Fig. 1b, c).

**Fig. 2:**
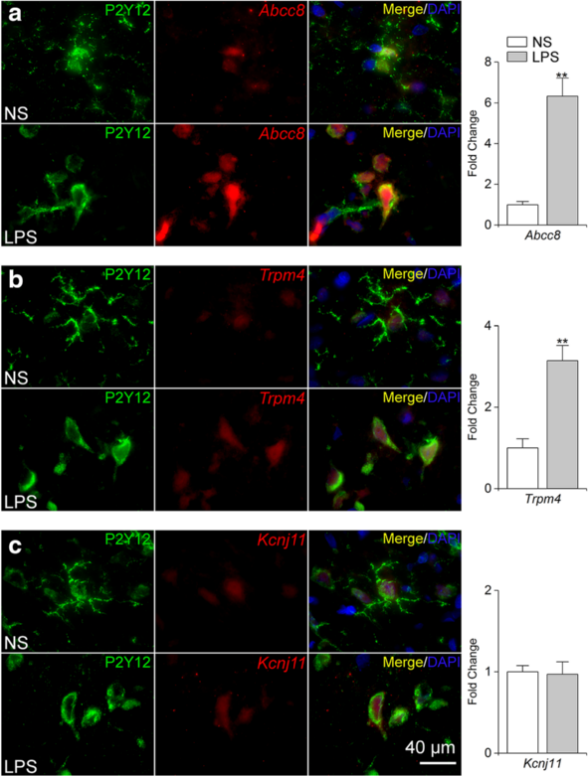
Upregulation of *Abcc8* and *Trpm4* mRNA in TLR4-activated rat microglia in vivo. **a**–**c** Immunofluorescence labeling of P2Y12^+^ microglia (*green*) and fluorescence in situ hybridization (FISH) of mRNA transcripts of *Abcc8, Trpm4*, or *Kcnj11* (*red*), following intrastriatal infusion (0.5 μL/h) of normal saline (*NS*) or *LPS* (5 μg/day) for 24 h (*LPS*); nuclei labeled with DAPI (*blue*); *bar graphs* quantification of microglial expression of *Abcc8, Trpm4*, or *Kcnj11*, expressed as fold change normalized to values with NS infusion; five replicates; **p < 0.01

Microglial expression of mRNA for *Abcc8*, *Trpm4*, and *Kcnj11*, the genes that express Sur1, Trpm4, and Kir6.2, was evaluated in vivo using combined in situ hybridization and immunofluorescence protein labeling in the same tissue section [54, 55], with P2Y12 immunolabeling used as the specific microglial marker. TLR4-activated P2Y12^+^ cells upregulated transcripts for *Abcc8* and *Trpm4* (fold increase of 6.33 ± 0.88 and 3.14 ± 0.37, respectively, *p* < 0.01; Fig. 2a, b); no change in expression of *Kcnj11* transcripts was observed (Fig. 2c).

**Fig. 3:**
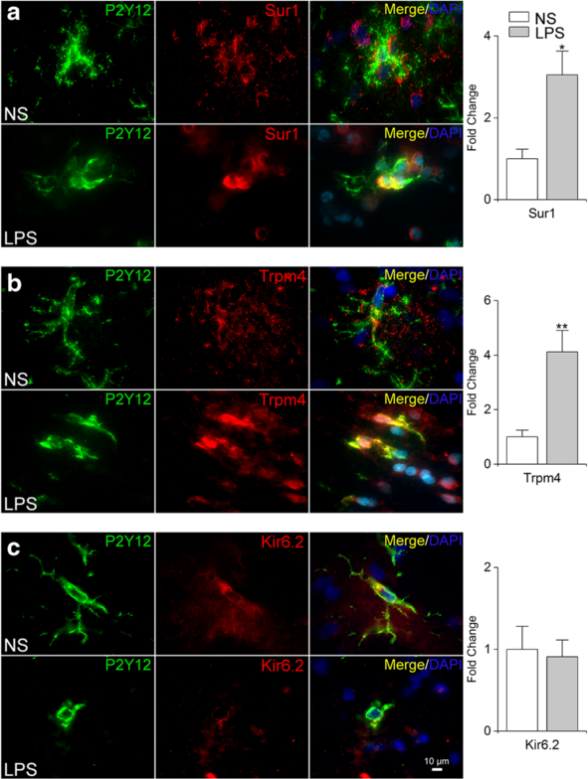
Upregulation of Sur1 and Trpm4 protein in TLR4-activated rat microglia in vivo. **a**–**c** Double immunofluorescence labeling of microglia (*P2Y12*, *green*) and Sur1, Trpm4, or Kir6.2 (*red*), following intrastriatal infusion (0.5 μL/h) of normal saline (*NS*) or LPS (5 μg/day) for 24 h (*LPS*); nuclei labeled with DAPI (*blue*); representative high magnification (×100) images are shown; *bar graphs* quantification of microglial expression of Sur1, Trpm4, or Kir6.2, expressed as fold change normalized to values with NS infusion; five replicates; *p < 0.05; **p < 0.01

Immunolabeling for Sur1, Trpm4, and Kir6.2 protein was evaluated in P2Y12^+^ cells following TLR4 activation (Fig. 3). TLR4-activated P2Y12^+^ cells exhibited enhanced immunolabeling for Sur1 and Trpm4 (fold increase of 3.05 ± 0.57 and 4.11 ± 0.78, respectively, *p* < 0.01; Fig. 3a, b). Consistent with a lack of *Kcnj11* induction, TLR4-activated P2Y12^+^ cells exhibited no change in Kir6.2 immunolabeling (Fig. 3c). Taken together, these data indicated that microglial activation by TLR4 in vivo resulted in the upregulation of mRNA and protein for the two subunits of the Sur1-Trpm4 channel, but not the pore-forming subunit of K_ATP_.

#### TLR4 activation induces Sur1-Trpm4 channel expression in primary cultured adult microglia

**Fig. 4:**
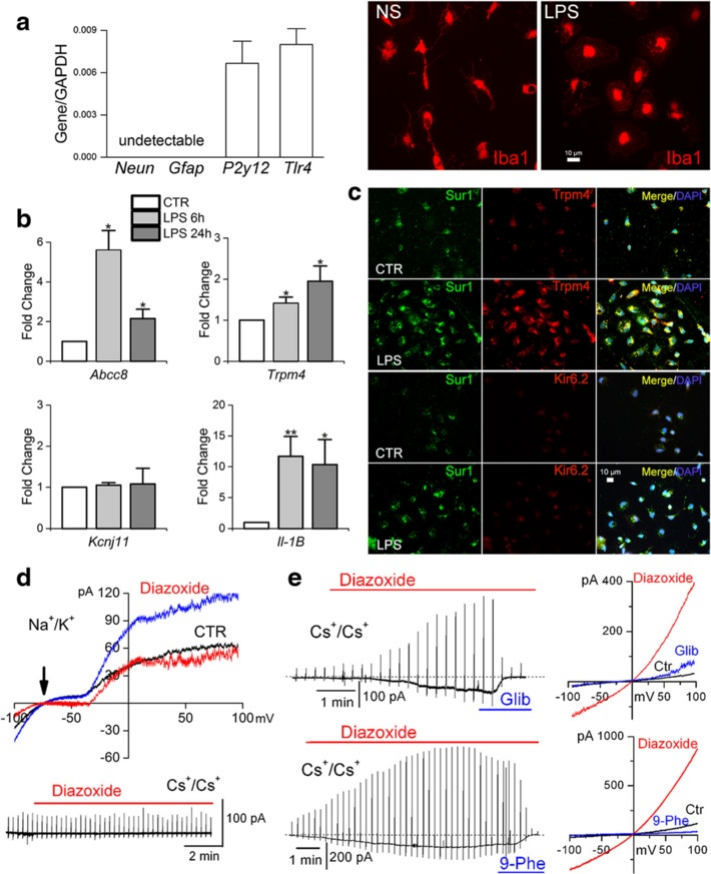
Upregulation of Sur1-Trpm4 channels in TLR4-activated primary rat adult microglia. **a** qPCR analysis of isolated cells (*left*) showing expression of microglial *P2y12* and *Tlr4*, and no expression of neuronal *Neun* or astrocytic *Gfap*; data are from three independent replicates; also shown are representative immunofluorescence images of Iba1^+^ isolated microglia under control conditions (normal saline, NS) versus 24 h after TLR4 ligation by LPS (1 μg/mL). **b** Fold change in mRNA for *Abcc8*, *Trpm4*, and *Kcnj11* in primary cultured adult microglia activated by ligation of TLR4 with LPS (1 μg/mL) for 24 h; induction of *I1-1β* mRNA was used as a positive control; ten replicates; * *p* < 0.05; ** *p* < 0.01. **c** Representative immunofluorescence images of primary cultured adult microglia showing expression of Sur1, Trpm4, and Kir6.2 protein under control (*CTR*) conditions and after ligation of TLR4 with LPS (1 μg/mL) for 24 h. **d** Whole-cell currents in quiescent primary cultured microglia recorded with physiological solutions (*upper*) and with Cs^+^-containing solutions (*lower*) during ramp pulses, shown at high (*upper*) and low (*lower*) temporal resolution; Sur1-activation by diazoxide yielded the difference current attributable to K_ATP_ (*red*). **e** Whole-cell currents in TLR4-activated primary cultured microglia recorded with Cs^+^-containing solutions during ramp pulses, shown at low (*left*) and high (*right*) temporal resolution, with Sur1-activation by diazoxide (100 μM), and blockade by glibenclamide (5 μM) (*upper*) or 9-phenanthrol (10 μM) (*lower*); the tracings in **d** and **e** are representative of six to eight cells per condition, with ramp pulses −100 to +100 mV in 500 ms, repeated every 15 s; holding potential, −50 mV

We used primary cultured adult rat microglia to determine if the induction of *Abcc8*/Sur1 and *Trpm4*/Trpm4 that we observed in vivo following TLR4 ligation was associated with the formation of functional Sur1-Trpm4 channels. Isolated cells were highly enriched for microglia (*P2y12r*^*+*^*/Tlr4*^*+*^ and *Gfap*^−^*/Neun*^−^) and reacted similarly to quiescent microglia in vivo, responding to TLR4 ligation with a shift in morphology from ramified to amoeboid (Fig. 4a).

Following TLR4 ligation, *Abcc8* mRNA was significantly upregulated at 6 and 24 h (5.6- and 2.2-fold vs. control, respectively, *p <* 0.05), as was mRNA for *Trpm4* (1.4- and 1.9-fold vs. control, respectively, *p <* 0.05) (Fig. 4b), corroborating our observations in vivo. mRNA for *Il-1β* also was significantly upregulated at both times (11.6- and 10.3-fold vs. control, respectively, *p <* 0.05). No change was observed in *Kcnj11* mRNA at either time. Consistent with the induction of *Abcc8* and *Trpm4*, enhanced immunolabelings for Sur1 and Trpm4 were observed 24 h after TLR4 activation, but no change in Kir6.2 immunoreactivity was apparent (Fig. 4c).

Sur1 forms heteromers not only with Kir6.2 (*Kcnj11*), but also with Kir6.1 (*Kcnj8*) [56, 57]. Following TLR4 ligation for 24 h, *Kcnj8* expression was decreased significantly (5.2-fold vs. control, *p* < 0.01; data not shown), suggesting that K_ATP_ comprised of Sur1-Kir6.1 is unlikely to play a role in TLR4-activated microglia.

Patch clamp recordings showed that quiescent primary rat microglia exhibited currents attributable to Sur1-Kir6.2 (K_ATP_) channels. Under basal conditions, currents recorded in physiological solutions (principal charge carriers, K^+^ intracellularly and Na^+^ extracellularly) showed both inward and delayed outward rectifier K^+^ currents that reversed at −75 mV, typical of primary microglia [58] (Fig. 4d, *CTR*). We used diazoxide to activate Sur1-regulated channels. Diazoxide activated a current that reversed at −75 mV, showed minimal conductance between the *E*_rev_ and −30 mV, and, above this, was outward, typical of K_ATP_ [59] (Fig. 4d, difference current in red). When Cs^+^-containing solutions (principal charge carrier, Cs^+^ intra- and extracellularly) were used, diazoxide failed to induce membrane currents in quiescent primary rat microglia (Fig. 4d, lower record). As Cs^+^ blocks K^+^ but not non-selective cation channels, these findings are consistent with the expression of K_ATP_ but not Sur1-Trpm4 channels in quiescent microglia.

By contrast, TLR4-activated microglia exhibited currents attributable to Sur1-Trpm4. Currents recorded in Cs^+^-containing solutions were activated by the Sur1 agonist, diazoxide, had a reversal potential of ~0 mV, and were blocked by both the Sur1 antagonist, glibenclamide, and the Trpm4 antagonist, 9-phenanthrol (Fig. 4e). In these cells, 5 μM glibenclamide or 10 μM 9-phenanthrol blocked >90 % of the diazoxide-induced inward current at −50 mV (seven and six cells, respectively).

Together, these findings indicated that TLR4 activation of microglia induces a switch from a quiescent phenotype expressing K_ATP_ channels to an activated phenotype with de novo upregulation of Sur1-Trpm4 channels.

#### TLR4 activation causes de novo Sur1-Trpm4 channel upregulation in N9 microglia

The N9 microglial cell line shares many phenotypic characteristics with primary microglia [60]. Here, we studied N9 microglia to determine whether they too would respond to TLR4 activation by upregulating Sur1-Trpm4 channels and to examine downstream signaling involving Sur1-Trpm4.

**Fig. 5:**
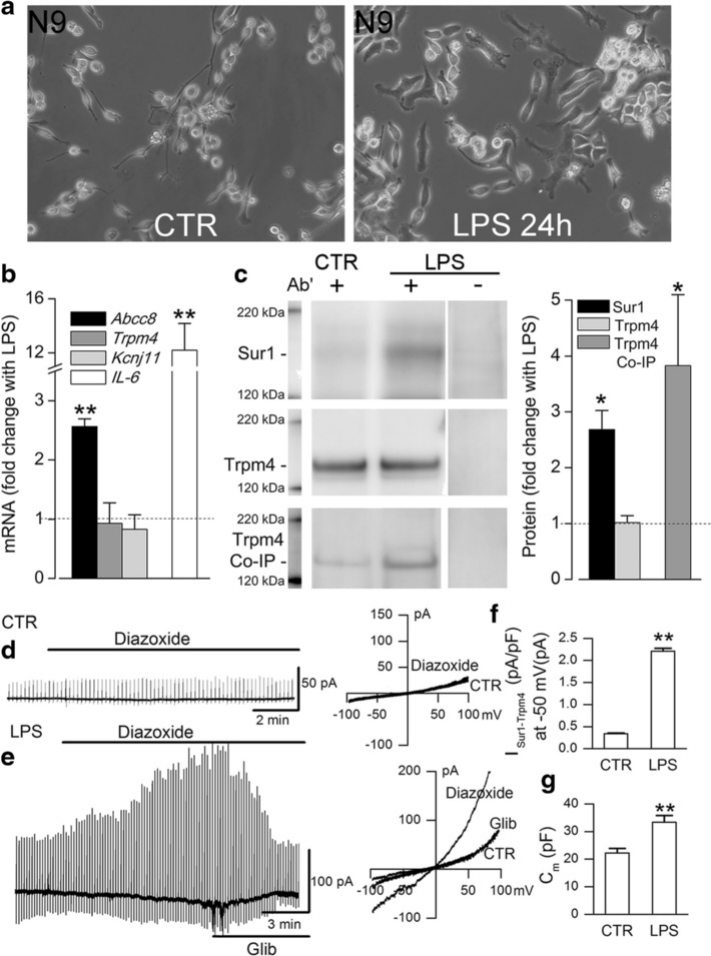
Upregulation of Sur1-Trpm4 channels in TLR4-activated murine N9 microglia. **a** Phase contrast images of N9 microglia under control (*CTR*) conditions (*left*) and 24 h after LPS treatment (1 μg/mL) (*right*). **b** Change in mRNA for *Abcc8*, *Trpm4*, and *Kcnj11* in N9 microglia activated by ligation of TLR4 with LPS (1 μg/mL) for 24 h; induction of *Il-6* mRNA was used as a positive control; six replicates; **p < 0.01; the *dotted line* indicates basal level of expression. **c** Immunoblots (*left panel*) and quantification (*right panel*) for Sur1 and Trpm4 of immunoisolates from N9 microglial lysates under control conditions (*CTR*) and following TLR4 activation for 24 h (*LPS*), with omission of IP antibody (*Ab′*) shown as a negative control; for co-immunoprecipitation (*Co-IP*), immunoisolation was performed using anti-Sur1 antibody and immunoblot was performed using anti-Trpm4 antibody; three replicates; *p < 0.05. **d**, **e** Whole-cell Cs^+^ currents at low (*left*) and high (*right*) temporal resolution during ramp pulses (−100 to +100 mV in 500 ms, repeated every 15 s; holding potential, −50 mV) in control N9 microglia (*CTR*) and N9 microglia after TLR4 activation by LPS (1 μg/mL) for 24 h; Sur1 was activated by diazoxide (100 μM) and inhibited by glibenclamide (5 μM). **f** Magnitude of the inward current density at −50 mV (*I*_Sur1-Trpm4_) activated by diazoxide in control versus TLR4-activated N9 microglia; same experiment as in **d** and **e**. **g** Magnitude of the membrane capacitance (*C*_m_) in control versus TLR4-activated N9 microglia; same experiment as in **d** and **e**; 14–16 cells/condition

As with primary cells, TLR4 activation of N9 microglia induced an activated phenotype heralded by a change in morphology to amoeboid (Fig. 5a). Following TLR4 activation for 24 h, *Abcc8* and *Il-6* mRNA were significantly elevated (fold change, 2.57 ± 0.13 and 12.2 ± 1.9, respectively, *p* < 0.05) (Fig. 5b). No change in mRNA abundance was observed for either *Trpm4* or *Kcnj11*.

Immunoprecipitation/immunoblot of whole cell lysate from N9 microglia showed minimal Sur1 expression under basal conditions and a significant increase in Sur1 following TLR4 activation (fold change, 2.68 ± 0.34, *p* < 0.05) (Fig. 5c). By contrast, basal expression of Trpm4 was prominent, and no change in Trpm4 expression was observed following TLR4 activation (Fig. 5c). Co-immunoprecipitation revealed a significant increase in co-associated Sur1 and Trpm4 following TLR4 activation (fold change, 3.83 ± 1.3, *p* < 0.05, Fig. 5c). Thus, in N9 cells, high basal levels of Trpm4 appeared to be sufficient for de novo formation of Sur1-Trpm4 following TLR4 activation.

Patch clamp recordings were used to determine whether co-assembled Sur1-Trpm4 heteromers formed functional channels in TLR4-activated N9 microglia. In quiescent N9 microglia, when Cs^+^ was used as the principal charge carrier, the Sur1 agonist, diazoxide failed to induce membrane currents, consistent with the absence of Sur1-regulated non-selective cation channels, and similar to findings in primary microglia. By contrast, TLR4-activated microglia exhibited Cs^+^ currents induced by diazoxide that had a reversal potential of ~0 mV and were blocked by the Sur1 antagonist, glibenclamide (Fig. 5d–f), consistent with Sur1-Trpm4 channels [41]. As expected, the change in microglial phenotype was accompanied by an increase in cell volume (Fig. 5g) [19].

Thus, TLR4 activation in N9 microglia resulted in a switch from a quiescent to an activated phenotype that was accompanied by de novo upregulation of Sur1-Trpm4 channels, similar to findings in primary microglia.

### Role of Sur1-Trpm4 in TLR4-activated microglia

#### Sur1-Trpm4 is a negative regulator of Ca^2+^ entry

**Fig. 6:**
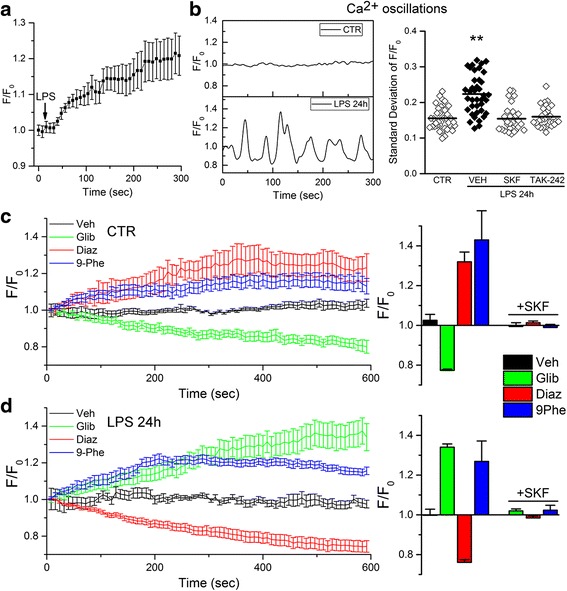
Sur1-Trpm4 is a negative regulator of SKF-96395-sensitive Ca^2+^ entry channels in murine N9 microglia. **a** Acute effect of LPS (1 μg/mL) on [Ca^2+^]_i_ in N9 microglia, expressed as fluorescence (*F*) over baseline (*F*_0_); data were obtained from ten cells per individual experiment; data shown are average responses of four independent replicates; the *black arrow* shows the time of LPS application. **b** Representative single cell traces of *F*/*F*_0_ in control (*CTR*) and TLR4-activated (*LPS* 24 h) N9 microglia (*left)*; also shown is the quantification of Ca^2+^ oscillations (*right*), expressed as the time series rolling standard deviation of *F*/*F*_0_, in CTR cells and in TLR4-activated cells treated with vehicle (*VEH*), the Ca^2+^ entry antagonist, SKF-96395 (*SKF*; 7.5 μM), or the TLR4 signaling inhibitor, TAK-242 (3 μM); **p < 0.01. **c**, **d** Temporal changes in [Ca^2+^]_i_, expressed as *F*/*F*_0_ (*left panels*), and magnitude of *F*/*F*_0_ at the termination of recording (*right panels*), in control (**c**) and N9 microglia after TLR4-activation by LPS (1 μg/mL) for 24 h (**d**), following application of vehicle (*Veh*), the Sur1 antagonist, glibenclamide (*Glib*; 30 μM), the Sur1 agonist, diazoxide (*Diaz*; 100 μM), the Trpm4 antagonist or 9-phenanthrol (*9Phe*; 5 μM); also shown is the magnitude of *F*/*F*_0_ after application of the Ca^2+^ entry antagonist, SKF-96395 (*SKF*; 7.5 μM) (*right panels*); the time of drug application was coincident with the start of recording; data were obtained from ten cells per individual experiment (*left*); average data collected at the end of 10 min recording from thee to five independent replicates are shown (*right*)

Numerous ligand-receptor interactions in microglia have been shown to give rise to Ca^2+^ influx [18, 20, 21], including LPS [50, 58, 61, 62]. Here, exposure of quiescent microglia to LPS induced an initial rise in [Ca^2+^]_i_ followed by plateau phase [58, 62] (Fig. 6a). Quiescent microglia were characterized by minimal dynamic changes in [Ca^2+^]_i_ over time, whereas microglia activated by TLR4 ligation for 24 h exhibited an oscillatory pattern of [Ca^2+^]_i_ (Fig. 6b, left panel, single cell traces), a phenomenon previously shown to depend on Trpm4 [63]. Ca^2+^ oscillation was abolished by TAK-242 inhibition of TLR4 signaling (Fig. 6b, right panel). LPS-induced increases in [Ca^2+^]_i_ [64, 65], as well as Ca^2+^ oscillation [66, 67], previously were shown to be mediated by SKF-96395-sensitive Ca^2+^ entry channels. Here, in N9 microglia, we found that LPS-induced oscillations also were abrogated by SKF-96395 (Fig. 6b, right panel).

In cells with Ca^2+^ entry mediated by non-voltage-operated ROCE and SOCE channels, Sur1-Kir6.2 (K_ATP_) channels on the one hand, and Trpm4 or Sur1-Trpm4 channels on the other hand, are expected to have opposite effects on Ca^2+^ entry, since opening of Sur1-Kir6.2 (K_ATP_) channels hyperpolarizes the cell membrane, whereas opening of Trpm4 or Sur1-Trpm4 depolarizes the cell membrane [26–31]. In quiescent microglia, diazoxide activation of Sur1 increased [Ca^2+^]_i_, and glibenclamide inhibition of Sur1 decreased [Ca^2+^]_i_ (Fig. 6c, red and green symbols). Opposite responses were found in TLR4-activated microglia, with diazoxide activation of Sur1 decreasing [Ca^2+^]_i_ and glibenclamide inhibition of Sur1 increasing [Ca^2+^]_i_ (Fig. 6d, red and green symbols). In both quiescent and TLR4-activated microglia, 9-phenanthrol inhibition of Trpm4 [68] increased [Ca^2+^]_i_ (Fig. 6c,d, blue symbols). Together, these findings are consistent with the interpretation that (i) both quiescent and activated N9 microglia express Trpm4 [58], which functions to limit Ca^2+^ influx [29, 69, 70]; (ii) quiescent microglia express Sur1-Kir6.2 (K_ATP_) channels [32–34], whose activation leads to hyperpolarization, which increases the inward driving force for Ca^2+^; and (iii) TLR4-activated microglia express Sur1-Trpm4 channels, whose activation leads to depolarization, which decreases the inward driving force for Ca^2+^ [26, 30].

#### Sur1-Trpm4 regulates NFATc1

The phenotype of N9 microglia is regulated by [Ca^2+^]_i_ and the transcription factor, NFAT, similar to TLR4-activated primary cultured microglia [71–73]. NFATc1 (NFAT2) is the isoform of NFAT that regulates the pro-inflammatory phenotype, including NOS2 expression, in activated microglia [14, 72]. NFAT is normally phosphorylated and sequestered in the cytoplasm. Nuclear translocation occurs following dephosphorylation by the Ca^2+^-sensitive phosphatase, calcineurin (CN) [74].

**Fig. 7:**
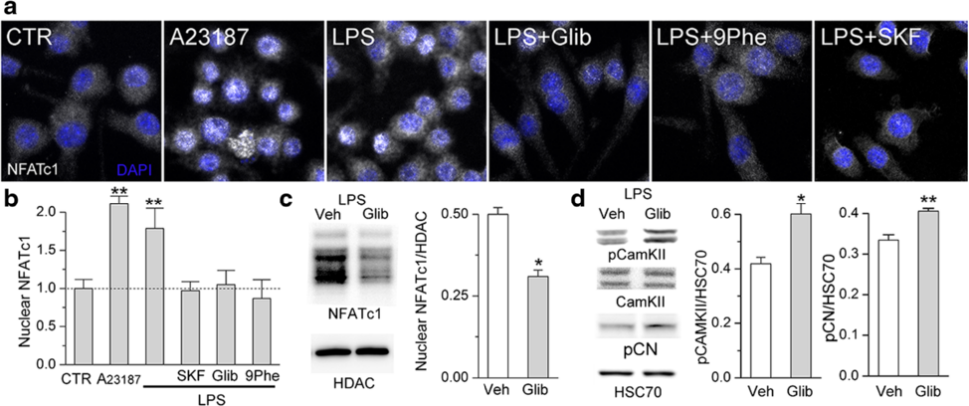
Sur1-Trpm4 regulates NFATc1, CaMKII, and CN in murine N9 microglia. **a**, **b** Images (**a**) and quantification (**b**) of nuclear NFATc1 (*white*) in N9 microglia under control (*CTR*) conditions, following 15-min exposure to the Ca^2+^ ionophore, A23187 (1 μM), used as positive control, and following 24-h exposure to LPS alone (1 μg/mL), LPS plus glibenclamide (*Glib*; 30 μM), LPS plus 9-phenanthrol (*9Phe*; 5 μM), or LPS plus SKF-96395 (*SKF*; 7.5 μM); nuclei stained with DAPI (*blue*); typical nuclear diameter is 6–12 μm; quantitative data on specific nuclear labeling were normalized to the control; *p* < 0.01. **c** Immunoblot (*left*) and quantification of all bands (*right*) for NFATc1 in nuclear extracts from TLR4-activated N9 microglia treated with vehicle (*Veh*) or glibenclamide (*Glib*; 30 μM);quantitative data were normalized to a loading control for the nuclear protein, histone deacetylase 1 (*HDAC*); four replicates. **d** Immunoblot (*left*) and quantification of all bands (*right*) for phosphorylated CaMKII (*pCaMKII*), *CaMKII*, and phosphorylated CN (*pCN*) in whole cell lysate from TLR4-activated N9 microglia treated with vehicle (*Veh*) or glibenclamide (*Glib*; 30 μM); quantitative data were normalized to a loading control for total protein, *HSC70*; four replicates; *p < 0.05

We evaluated NFATc1 activation in N9 microglia (Fig. 7a–c). Control cells were characterized by NFATc1 immunoreactivity that was confined mostly to the cytoplasm. Increasing [Ca^2+^]_i_ using the Ca^2+^ ionophore, A23187, significantly increased nuclear NFATc1. TLR4 activation for 24 h, which increases [Ca^2+^]_i_ (Fig. 6a,b), also induced nuclear translocation of NFATc1 [63]. In the presence of LPS, inhibition of Ca^2+^ influx by SKF-96395, which previously was shown to inhibit NFATc3 nuclear translocation [75], here was shown to inhibit NFATc1 nuclear translocation.

Since inhibition of Sur1-Trpm4 by glibenclamide or 9-phenanthrol increases [Ca^2+^]_i_ (Fig. 6d), and since increasing [Ca^2+^]_i_ activates NFAT, we expected that Sur1-Trpm4 channel inhibition would increase NFAT nuclear translocation. Unexpectedly, glibenclamide and 9-phenanthrol inhibited LPS-induced nuclear translocation of NFATc1 (41 and 51 % reduction vs. LPS alone) (Fig. 7a, b). Nuclear immunoblots confirmed that glibenclamide inhibition of Sur1-Trpm4 reduced the nuclear accumulation of NFATc1 after TLR4 activation (38 % reduction vs. LPS alone, Fig. 7c).

#### Sur1-Trpm4 inhibition activates CaMKII in microglia

One possible explanation for the unexpected finding that glibenclamide inhibition of Sur1-Trpm4 reduced nuclear accumulation of NFATc1 is that the increase in [Ca^2+^]_i_ induced by channel blockade caused activation of CaMKII. In vascular smooth muscle cells and cardiac myocytes, increasing [Ca^2+^]_i_ activates not only CN but also CaMKII, with higher levels of Ca^2+^/calmodulin being required to activate CaMKII, compared to CN [16, 76]. Importantly, activated CaMKII negatively regulates NFAT signaling by phosphorylating CN at Ser197, which inhibits its phosphatase activity [76], thus allowing NFAT to be more phosphorylated and so maintained within the cytoplasm [16, 76, 77]. Here, we found that in TLR4-activated N9 microglia, glibenclamide inhibition of Sur1-Trpm4, which we showed previously augments [Ca^2+^]_i_, led to a significant increase in phosphorylated CaMKII (pCaMKII) (30 % increase vs. LPS alone, Fig. 7d) and a significant increase in phosphorylated CN (pCN) (23 % increase vs. LPS alone, Fig. 7d), consistent with the reduced nuclear translocation of NFATc1 observed with glibenclamide.

#### Sur1-Trpm4 regulates binding of NFATc1 to the Nos2 promoter

**Fig. 8:**
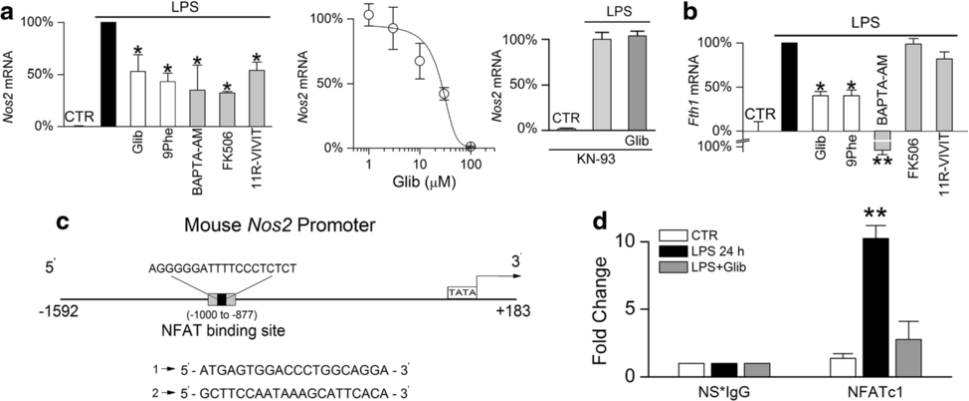
Sur1-Trpm4 regulates the binding of NFATc1 to the *Nos2* promoter in murine N9 microglia. **a** Percent change in mRNA for *Nos2* in N9 microglia (*left*) under control (CTR) conditions, or following 24-h exposure to LPS alone (1 μg/mL), LPS plus glibenclamide (*Glib*; 30 μM), LPS plus 9-phenanthrol (*9Phe*; 5 μM), LPS plus BAPTA-AM (10 μM), LPS plus FK506 (1 μM), or LPS plus 11R-VIVIT (10 μM); data normalized to values with LPS alone; five replicates; *p < 0.05; also shown (*middle*) is the concentration-response relationship for LPS induction of *Nos2* mRNA versus glibenclamide concentration; EC_50_, 26 μM; experiments performed in 5 % fetal bovine serum; three replicates; also shown (*right*) is the absence of effect of glibenclamide on *Nos2* mRNA induction in the presence of the CaMKII inhibitor, *KN-93*; three replicates. **b** Percent change in mRNA for *Fth1* in N9 microglia under control (*CTR*) conditions or following 24-h exposure to LPS alone (1 μg/mL), LPS plus glibenclamide (*Glib*; 30 μM), LPS plus 9-phenanthrol (*9Phe*; 5 μM), LPS plus BAPTA-AM (10 μM), LPS plus FK506 (1 μM), or LPS plus 11R-VIVIT (10 μM); data normalized to values with LPS alone, which represented a four to fivefold increase; five replicates; *p < 0.05; **p < 0.01. **c** Schematic of the mouse *Nos2* promoter; gray box indicates the region from –1000 to –877 used for PCR, with sequences of primers used to amplify immunoisolated DNA shown below; this region of the promotor contains an NFAT binding site (*black box*; sequence shown above). **d** Quantification of immunoisolated DNA following ChIP with an anti-NFATc1 antibody or species-matched non-specific IgG (NS*IgG) from N9 microglia under control (*CTR*) conditions or following 24-h exposure to LPS alone (1 μg/mL) or LPS plus glibenclamide (*Glib*; 30 μM); three replicates; **p < 0.01

NFATc1 is a key transcriptional regulator of *Nos2* gene expression. Strong induction of *Nos2* mRNA was observed in TLR4-activated microglia (Fig. 8a). As reported [61], treatment with the Ca^2+^ chelator, BAPTA-AM, significantly attenuated *Nos2* induction (66 % reduction versus LPS alone). Inhibition of NFAT, either indirectly by FK506 inhibition of CN, or directly by 11R-VIVIT [72, 78], significantly reduced the induction of *Nos2* mRNA (68 and 46 % reduction, respectively).

Inhibition of Sur1 by glibenclamide dose dependently reduced *Nos2* mRNA induction in TLR4-activated microglia, with an EC_50_ of 26 μM (Fig. 8a). An MTT cell viability assay showed that glibenclamide was not cytotoxic across the range of concentrations tested (data not shown). Since these experiments were carried out in the presence of 5 % FBS, and since glibenclamide is 99 % protein bound [48], these data suggest an apparent EC_50_ value of ~260 nM free glibenclamide. The Trpm4 inhibitor, 9-phenanthrol, also significantly reduced *Nos2* mRNA induction in TLR4-activated microglia (57 % reduction) (Fig. 8a). Notably, the inhibition of *Nos2* mRNA induction by glibenclamide was prevented by blockade of CaMKII with KN-93 (Fig. 8a). Thus, the TLR4-mediated induction of *Nos2* mRNA was sensitive to [Ca^2+^]_i_, NFAT, and Sur1-Trpm4, and the effect of Sur1 inhibition was dependent on CaMKII.

As a control, we evaluated the induction of ferritin H mRNA (*Fth1*), an inducible iron binding protein whose expression is Ca^2+^-dependent but NFAT-independent [79]. Similar to *Nos2*, *Fth1* mRNA was strongly induced in TLR4-activated microglia and was sensitive to BAPTA-AM chelation of Ca^2+^ (Fig. 8b). In further support of Sur1-Trpm4 regulation of Ca^2+^-dependent transcriptional mechanisms, both glibenclamide and 9-phenanthrol inhibition of Sur1-Trpm4 significantly reduced the induction of *Fth1* mRNA in TLR4-activated microglia, although inhibition of NFAT by either FK506 or 11R-VIVIT had no effect (Fig. 8b).

ChIP experiments were carried out to determine whether the reduction of *Nos2* induction resulting from Sur1-Trpm4 inhibition was due to reduced binding of NFATc1 to the *Nos2* promoter. We independently verified the sequence of an NFAT binding site on the *Nos2* promoter [14] (Fig. 8c). ChIP was performed from intact N9 cells using antibodies directed against NFATc1, with species-matched non-specific IgG (NS*IgG) used as a control. qPCR analysis of the immunoprecipitates was performed using primers generated to the −1000 to −877 region spanning the NFAT binding site. ChIP showed that LPS increased NFATc1 binding to the *Nos2* promoter (Fig. 8d), consistent with TLR4 activation inducing NFAT-dependent *Nos2* mRNA expression. Moreover, glibenclamide inhibition of Sur1-Trpm4 significantly attenuated NFATc1 binding to the *Nos2* promoter (Fig. 8d), consistent with Sur1-Trpm4 negatively regulating NFATc1. Together, these data indicated that inhibition of Sur1-Trpm4 decreases *Nos2* induction in TLR4-activated N9 microglia due to a reduction in NFATc1 binding to the *Nos2* promoter.

#### Sur1-Trpm4 regulates induction of NOS2 protein and nitrite production

**Fig. 9:**
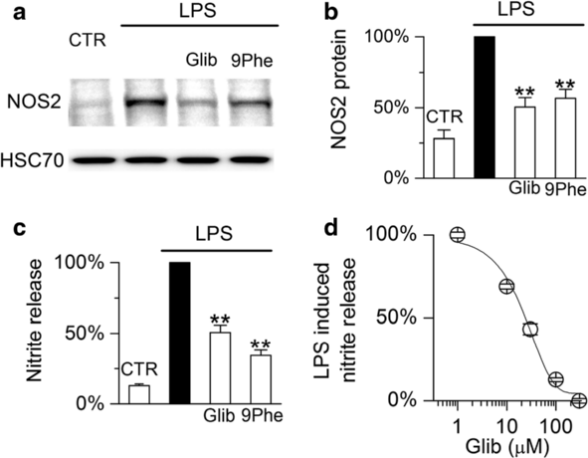
Sur1-Trpm4 regulates induction of NOS2 protein in murine N9 microglia. **a**, **b** Immunoblot (**a**) and densitometric analysis (**b**) for NOS2 protein in N9 microglia under control (*CTR*) conditions or following 24-h exposure to LPS alone (1 μg/mL), LPS plus glibenclamide (*Glib*; 30 μM), or LPS plus 9-phenanthrol (*9Phe*; 5 μM); quantitative data were normalized to a loading control for total protein, *HSC70*, and to values with LPS alone; five replicates; **p < 0.01. **c** Percent nitrite in the medium of N9 microglial cultures under control (*CTR*) conditions or following 24-h exposure to LPS alone (1 μg/mL), LPS plus glibenclamide (*Glib*; 30 μM), or LPS plus 9-phenanthrol (*9Phe*; 5 μM); nitrite was measured using the Griess assay; data normalized to values with LPS alone, with maximum nitrite ranging from 8 to 13 μM per experiment; three replicates. **d** Concentration-response relationship for LPS-induced nitrite in the medium versus glibenclamide; EC_50_, 24 μM; experiments performed in 5 % fetal bovine serum; three replicates

Significantly less expression of NOS2 protein was observed in activated microglia treated with either glibenclamide or 9-phenanthrol (50 and 44 % reduction, respectively) (Fig. 9a, b), in agreement with Sur1-Trpm4 inhibition reducing *Nos2* mRNA induction.

We also studied the effect of Sur1-Trpm4 inhibition on nitrite production, a functional measure of NOS2 activity, using the Griess assay. Both glibenclamide and 9-phenanthrol significantly attenuated nitrite production in TLR4-activated microglia (Fig, 9c), consistent with Sur1-Trpm4 inhibition reducing TLR4-mediated upregulation of *Nos2*/NOS2. The effect of glibenclamide on nitrite production was dose dependent, with an EC_50_ value similar to that observed for *Nos2* mRNA induction (Fig. 9d).

#### Abcc8−/− and Trpm4−/− protects against TLR4-mediated Nos2 induction in vivo

**Fig. 10:**
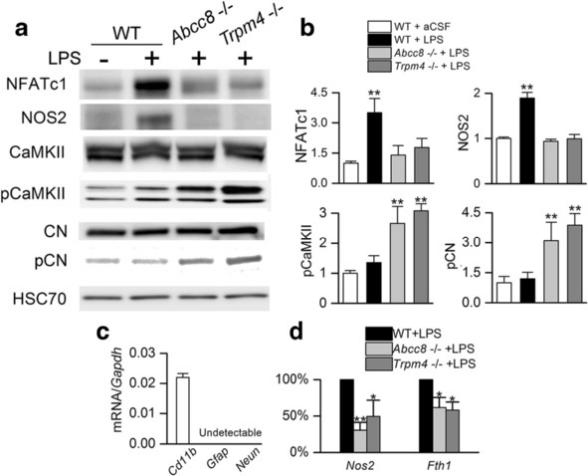
*Abcc8*−/− and *Trpm4*−/− protects against TLR4-mediated NOS2 induction in murine microglia. **a**, **b** Immunoblots (**a**) and quantification (**b**) for NFATc1 (90 kDa isoform shown), NOS2 (130 kDa), pCaMKII (α/β, 50/60 kDa), CaMKII (α/β, 50/60 kDa), pCN (60 kDa), and CN (60 kDa), for tissue homogenates from the striatum of wild-type (*WT*), *Abcc8*−/− and *Trpm4*−/− mice, 24 h after injection of aCSF (5 μL) or LPS (5 μL; 0.1 μg/μL) into the striatum; gels were run separately for each protein analyzed; quantitative data were normalized to a loading control for total protein, *HSC70*; three replicates; **p < 0.01. **c** Quantification of mRNA for microglial *Cd11b*, astrocytic *Gfap*, and neuronal *Neun* in freshly isolated microglia from mouse brain; data normalized to mRNA for *Gapdh*; three replicates. **d** Quantification of mRNA induction for *Nos2* and *Fth1* in primary cultured adult microglia from WT, *Abcc8*−/− and *Trpm4*−/− mice 24 h after exposure to LPS (1 μg/mL); data normalized to the response of LPS alone in WT microglia; three replicates; *p < 0.05; **p < 0.01

The results of the foregoing experiments predict that gene silencing of *Abcc8* and of *Trpm4* should protect against TLR4 activation in vivo. To test this hypothesis, sterile aCSF or LPS was injected into the striatum of adult WT, *Abcc8*−/− and *Trpm4*−/− mice to study the effect of TLR4 activation. Basal expression of CaMKII, CN, NFATc1, and NOS2 were similar in naïve (no injection) WT, *Abcc8*−/− and *Trpm4*−/− mice (data not shown). In WT mice, TLR4 activation increased NFATc1, consistent with NFATc1-mediated auto-upregulation of *Nfatc1* [80] and upregulation of NOS2 expression. By contrast, in *Abcc8*−/− and *Trpm4*−/− mice, NFATc1 and NOS2 upregulation were significantly impaired (Fig. 10a, b), recapitulating the effect of glibenclamide in N9 microglia (Fig. 7a–c and Fig. 9).

Total levels of CaMKII and CN were unaffected by TLR4 ligation (Fig. 10a). LPS injection in WT mice resulted in minimal changes in pCaMKII and pCN, but in *Abcc8*−/− and *Trpm4*−/− mice, LPS injection resulted in significant increases in pCaMKII and pCN (Fig. 10a, b). These findings accord with the reduced levels of NFATc1 and NOS2 expression in these genotypes and recapitulate the effect of glibenclamide in N9 microglia (Fig. 7d).

Finally, to determine whether the in vivo findings in the different murine genotypes were attributable to microglia, we evaluated the induction of *Nos2* and *Fth1* mRNA in primary cultured microglia isolated from adult WT, *Abcc8*−/−, and *Trpm4*−/− mice. Isolated cells were highly enriched in microglia (*Cd11b*), with no detectable for astrocytes (glial fibrillary acidic protein (*Gfap*)) or neurons (*Neun*) (Fig. 10c). TLR4-mediated induction of both *Nos2* and *Fth1* mRNA was significantly reduced in microglia derived from *Abcc8*−/− mice, by 70 and 39 %, respectively, and from *Trpm4*−/− mice, by 49 and 42 %, respectively (Fig. 10d), recapitulating the effect of glibenclamide and 9-phenanthrol in N9 microglia (Fig. 8).

## Discussion

**Fig. 11:**
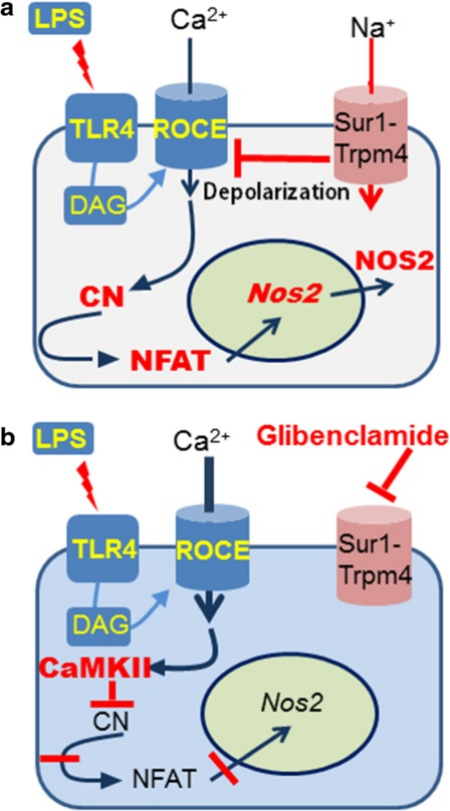
Model of Sur1-Trpm4 regulation of Ca^2+^ entry, NFAT and *Nos2*/NOS2 in TLR4-activated microglia. **a**, **b** Depiction of NFAT-mediated expression of *Nos2*/NOS2 in TLR4-activated microglia under normal conditions (**a**) and following inhibition of Sur1-Trpm4 by glibenclamide (**b**). Sur1-Trpm4 normally acts, via membrane depolarization, to regulate Ca^2+^ entry via SKF-96395-sensitive channels (e.g., ROCE), leading to activation of calcineurin (*CN*) and nuclear factor of activated T cells (*NFAT*), resulting in expression of *Nos2*/NOS2. When Sur1-Trpm4 is blocked by glibenclamide, excess Ca^2+^ enters the cell, preferentially activating CaMKII, which inhibits CN/NFAT and reduces the expression of *Nos2*/NOS2

The major findings of the present study are that (i) TLR4-activated microglia exhibit de novo upregulation of Sur1-Trpm4 channels; (ii) microglial Sur1-Trpm4 channels act as negative regulators of SKF-96395-sensitive Ca^2+^ entry channels; and (iii) whereas normally, TLR4 activation causes preferential activation of CN/NFATc1, resulting in induction of *Nos2*/NOS2, silencing or pharmacological blockade of *Abcc8*/Sur1 or *Trpm4*/Trpm4 causes preferential activation of CaMKII, resulting in reduced NFAT activation and reduced induction of *Nos2*/NOS2. Our finding that *Nos2*/NOS2 induction is reduced via pCaMKII-mediated inhibition of CN/NFATc1 in *Abcc8*−/− and *Trpm4*−/− mice constitutes a novel mechanism of ion channel-mediated control of Ca^2+^-dependent gene regulation [81, 82] (summarized in Fig. 11).

We validated N9 microglia as a tool to study the role of Sur1-Trpm4 in TLR4-activated microglia. This was important because cell signaling is difficult to study in primary microglial cultures, due to their relatively limited number, whereas microglial cell lines, which provide sufficient quantities of cells for cell signaling studies, cannot be assumed a priori to function identically to primary microglia [60, 83]. There were several notable similarities between N9 cells and primary adult microglia following TLR4 activation: (i) the transition from a quiescent to an activated morphology; (ii) no induction of *Kcnj11*/Kir6.2; (iii) induction of *Abcc8*/Sur1; (iv) de novo upregulation of functional Sur1-Trpm4 channels; (v) LPS-induced acute elevation of [Ca^2+^]_i_ and oscillatory Ca^2+^ signaling [50, 58]; and (vi) NFAT-dependent regulation of the pro-inflammatory phenotype [71–73]. A notable difference between N9 microglia and primary adult microglia was the lack of induction of *Trpm4*/Trpm4 in N9 cells by TLR4 activation. However, high basal expression of Trpm4 in N9 cells, which was not present in primary cells, may have masked or precluded *Trpm4*/Trpm4 induction. Despite this difference, TLR4 activation in both N9 microglia and in primary adult microglia resulted in de novo upregulation of Sur1-Trpm4 channels. In addition, the effects of Sur1-Trpm4 inhibition or silencing on downstream TLR4 signaling were similar in N9 microglia, in primary cultured murine microglia, and in mouse brain. In all cases, NFAT activation and *Nos2*/NOS2 induction were markedly decreased by channel inhibition, even though the effects on pCN and pCaMKII appeared less robust in the N9 cells compared to the primary cells and tissues (Fig. 7 versus 10).

Ortega and colleagues [32] were the first to report an effect of glibenclamide on microglial activation in a rodent model of ischemic stroke. Utilizing the BV2 microglial cell line, they reported induction of *Abcc8*/Sur1 and *Kcnj11*/Kir6.2 following exposure to LPS + IFNγ for 48 h, as well as enhanced immunolabeling of microglia in vivo for subunits of Sur1-Kir6.2 (K_ATP_) in cerebral ischemia. In their reports [32–34], they attributed the beneficial effects of glibenclamide in cerebral ischemia to inhibition of Sur1-Kir6.2 (K_ATP_). Here, studying microglia in vivo and primary cultured adult microglia in vitro, we observed currents in quiescent microglia that were clearly attributable to K_ATP_ channels, and we confirmed the observations of Ortega et al. on *Abcc8*/Sur1 upregulation following TLR4 activation. However, we did not observe *Kcnj11*/Kir6.2 upregulation in any of our experiments with TLR4 activation, similar to Virgili et al. [84], who found no change in Kir6.2 in BV2 microglia or in primary cultured murine microglia exposed to LPS + IFNγ. Instead, we found that TLR4 activation induced de novo expression of Sur1-Trpm4 channels in both primary cultured adult microglia and in N9 cells.

In TLR4-activated microglia, the dominant Sur1-regulated channel appears to be Sur1-Trpm4, not Sur1-Kir6.2. First, we showed that Sur1-activation by diazoxide has opposite effects in TLR4-activated microglia compared to quiescent microglia. In quiescent microglia, Sur1 activation *increases* Ca^2+^ influx, consistent with Sur1-Kir6.2 activation hyperpolarizing the cells and increasing the inward driving force for Ca^2+^. In TLR4-activated microglia, Sur1 activation *decreases* Ca^2+^ influx, consistent with Sur1-Trpm4 activation depolarizing the cells and decreasing the inward driving force for Ca^2+^. Second, blockade of Sur1 in TLR4-activated cells increases Ca^2+^ and increases pCaMKII, consistent with involvement of Sur1-Trpm4 and ROCE/SOCE channels. Blockade of Sur1-Kir6.2 could explain the latter finding, but only if VOCE channels were mediating Ca^2+^ influx. However, Trpm4 blockade, which hyperpolarizes the cell and should deactivate VOCE channels, led to an increase, not a decrease in [Ca^2+^]_i_, consistent with the absence of involvement of VOCE channels. The most parsimonious explanation for our combined observations is that Sur1-Trpm4 channels are the dominant contributors to the effects of Sur1-modulation in TLR4-activated microglia. Sur1-Kir6.2 channels also may present, but the net effects of Sur1-modulation in TLR4-activated microglia appear to be determined by Sur1-Trpm4.

Alterations in Ca^2+^ homeostasis contribute to microglia-mediated progression of CNS disorders [19, 20, 61, 85, 86]. Regulation of [Ca^2+^]_i_ is critical for the initiation and maintenance of distinct transcriptional programs underlying potentially harmful microglial phenotypes [18–20]. Our data indicate that Sur1-Trpm4 channels are an important mechanism for regulating Ca^2+^ entry and downstream Ca^2+^-signaling in TLR4-activated microglia. Sur1-Trpm4 channels are activated by intracellular Ca^2+^, with a rise in [Ca^2+^]_i_ linking directly to membrane depolarization, providing negative feedback that opposes additional Ca^2+^ entry. Co-assembly with Sur1 increases the apparent sensitivity of Trpm4 to intracellular Ca^2+^, thereby strengthening Trpm4’s role as a negative regulator of Ca^2+^ entry [41]. The Sur1-Trpm4 channel thus may be an important treatment target in degenerative diseases of the CNS mediated by TLR4-activated microglia.

Calcineurin is a critical mechanism by which activated microglia shape their response to TLR4-ligation and control their phenotype [19, 87]. The activation of CN/NFAT depends on the amplitude and duration of Ca^2+^ signals in combination with other Ca^2+^-dependent signals that may provide negative feedback [88, 89]. NFAT is said to function as a “working memory” of Ca^2+^ signaling that is more efficiently activated by low-amplitude, repetitive oscillations in [Ca^2+^]_i_ than by continuous Ca^2+^ influx [88, 90]. In accord with this, we observed that TLR4 activation for 24 h in N9 microglia led to oscillations of [Ca^2+^]_i_ accompanied by manifestations of the activated phenotype, including morphological changes, Sur1-Trpm4 upregulation, and *Nos2*/NOS2 induction.

The paradoxical observations that inhibition of Sur1-Trpm4 caused an elevation in [Ca^2+^]_i_ but that it significantly reduced activation of NFATc1 led us to consider alternative Ca^2+^-dependent mechanisms regulating CN. Importantly, sustained elevations of [Ca^2+^]_i_ result in autonomous, persistent activation of CaMKII [77]. When CaMKII activity is augmented in vascular smooth muscle cells or in cardiac myocytes, the effects of increased Ca^2+^ on NFAT nuclear translocation are significantly attenuated, due to direct inhibition of CN [16, 76]. CaMKII-dependent processes were described recently in microglia [91], although CaMKII regulation of CN was not investigated. Inhibition/gene suppression of Sur1-Trpm4 following TLR4 activation resulted in significant increases in phosphorylated CaMKII (Fig. 7d) and in phosphorylated CN (Fig. 7d), consistent with this mechanism accounting for the attenuated NFATc1 translocation and reduced *Nos2*/NOS2 induction that we observed.

Glibenclamide is not the only treatment to reduce NOS2 expression by activated microglia. Pretreatment of primary cultured neonatal microglia or BV2 cells with diazoxide prior to exposure to LPS + IFNγ reduces NOS2 expression and nitrite production [84, 92]. Since diazoxide *opens* Sur1-regulated channels, whereas glibenclamide inhibits the same channels, our findings, as reported here, may seem to contradict published findings. However, our data showing that pharmacological inhibition of Sur1 reduces NOS2 were confirmed by similar results obtained with genetic inhibition of Sur1 via silencing of *Abcc8*, both in vivo and in vitro. Notably, the molecular mechanism proposed for the anti-inflammatory effect of diazoxide involves a general reduction in the overall microglial response to activation signals [93], whereas the molecular mechanism that we propose for the anti-inflammatory effect of glibenclamide involves blockage of CN/NFAT-signaling *after* microglial activation (Fig. 11). Thus, Sur1-active drugs with different mechanisms of action may affect different aspects of the overall microglial inflammatory response, yet bring about a similar endpoint.

An important property of the Sur1-Trpm4 channel is that both subunits, Sur1 and Trpm4, are required for the manifestation of its pathological effects. This pathognomonic property was first described in an animal model of traumatic spinal cord injury, where pharmacological blockade of Sur1 (glibenclamide, repaglinide) or of Trpm4 (flufenamic acid, riluzole), gene suppression (antisense oligodeoxynucleotide against *Abcc8* or *Trpm4*), and gene silencing (*Abcc8−/−* or *Trpm4*−/−), all were shown to result in exactly the same phenotype—educed microvascular dysfunction and capillary fragmentation [94]. Similarly, in a murine model of experimental autoimmune encephalomyelitis, silencing of *Abcc8* or of *Trpm4* results in the same phenotype, with reduced neuroinflammation and preservation of white matter [36, 95]. Our present findings extend these previous observations, showing that in TLR4-mediated neuroinflammation, silencing *Abcc8* or *Trpm4* results in the same phenotype—preferential activation of CaMKII over CN/NFATc1 and reduced induction of *Nos2*/NOS2.

Our findings indicate that Trpm4 is the major molecular partner of Sur1 following TLR4 activation in microglia and that the beneficial effects of glibenclamide in the setting of TLR4-induced neuroinflammation may be due, in part, to augmented CaMKII signaling in microglia. Blockade of Sur1-Trpm4 by glibenclamide previously was shown to be protective in models of ischemic and traumatic CNS injury, where the activity of Sur1-Trpm4 in neurons, astrocytes, and endothelial cells can result in excess Na^+^ influx leading to catastrophic cell swelling [26]. In microglia, however, the activity of Sur1-Trpm4 is deleterious for a different reason—namely, it aids in the dynamic regulation of [Ca^2+^]_i_ that is required for a sustained neuroinflammatory response.

## Conclusions

Sur1-Trpm4 channels constitute a novel mechanism by which TLR4-activated microglia regulate pro-inflammatory, Ca^2+^-sensitive gene expression, including *Nos2*/NOS2. Glibenclamide blockade of Sur1-Trpm4 is promising for the future treatment of CNS diseases involving neuroinflammation and nitrosative/oxidative stress.

## Abbreviations

[Ca^2+^]_i_, intracellular concentration of calcium; aCSF, artificial cerebrospinal fluid; ANOVA, analysis of variance; BAPTA-AM, 1,2-bis(2-Aminophenoxy)ethane-*N*,*N*,*N*′,*N*′-tetraacetic acid acetoxymethyl ester; CaMKII, Ca^2+^/calmodulin protein kinase II; Cd11b, cluster of differentiation molecule 11b; CD68/ED1, cluster of differentiation molecule 68; CHAPS, 3-[(3-cholamidopropyl)dimethylammonio]-1-propanesulfonate; ChIP, chromatin immunoprecipitation; CN, calcineurin; CNS, central nervous system; DAMP, danger-associated-molecular pattern; DAPI, 4′,6-diamidino-2-phenylindole; DIG, digoxigenin; DMEM, Dulbecco’s modified Eagle’s medium; DMSO, dimethylsulfoxide; FBS, fetal bovine serum; Gapdh, glyceraldehyde 3-phosphate dehydrogenase; Gfap, glial fibrillary acidic protein; HBSS, Hank’s balanced salt solution; HDAC1, histone deacetylase 1; Iba1, ionized Ca^2+^-binding adapter molecule 1; IMDM, Iscove’s modified Dulbecco’s medium; IP, immunoprecipitation; ISH, in situ hybridization; K_ATP_, ATP-sensitive potassium channel; LDH, lactate dehydrogenase; LPS, lipopolysaccharide; NFAT, nuclear factor of activated T-cells; NO, nitric oxide; NOS2, inducible nitric oxide synthase; NS, normal saline; PPI, protease and phosphatase inhibitor cocktail; qPCR, quantitative real-time polymerase chain reaction; RIPA, radio-immunoprecipitation assay; ROCE, receptor-operated Ca^2+^ entry; ROI, region of interest; SDS-PAGE, sodium dodecyl sulfate polyacrylamide gel electrophoresis; SIP, standard isotonic Percoll; SOCE, store-operated Ca^2+^ entry; Sur1, sulfonylurea receptor 1; TLR4, Toll-like receptor 4; Trpm4, transient receptor potential melastatin 4; VOCE, voltage-operated Ca^2+^ entry; WT, wild type
